# Correction: Basal ganglia role in learning rewarded actions and executing previously learned choices: Healthy and diseased states

**DOI:** 10.1371/journal.pone.0298878

**Published:** 2024-02-08

**Authors:** Garrett Mulcahy, Brady Atwood, Alexey Kuznetsov

There are errors in the Funding section. The correct Funding statement is: A. Kuznetsov acknowledges the support from the grant P60 AA007611.
